# Non-invasive diagnostic potential of salivary miR-25-3p for periodontal disease and osteoporosis among a cohort of elderly patients with type 2 diabetes mellitus

**DOI:** 10.1186/s12903-023-02992-2

**Published:** 2023-05-23

**Authors:** Jing Ni, Qiong Zhang, Fei Lei

**Affiliations:** 1 Clinical Medicine, Xi’an Medical University, Xi’an, China; 2 Department of Endocrinology, The Second Affiliated Hospital of Xi’an Medical University, Xi’an, China; 3 Department of Stomatology, The Second Affiliated Hospital of Xi’an Medical University, No. 167, Fangdong Street, Baqiao District, Xi’an, 710038 Shaanxi China

**Keywords:** Type 2 diabetes mellitus, Osteoporosis, Periodontal disease, miR-25-3p, Saliva

## Abstract

**Objective:**

Osteoporosis (OP) and periodontal disease (PD) are two common health issues that threaten the older population and potentially connected each other in the context of type 2 diabetes mellitus (T2DM). Dysregulated expression of microRNAs (miRNAs) may contribute to the development and progression of both OP and PD among elderly T2DM patients. The present study aimed to evaluate the accuracy of miR-25-3p expression for the detection of OP and PD when compared to a mixed group of patients with T2DM.

**Methods:**

The study recruited 45 T2DM patients with normal bone mineral density (BMD) and healthy periodontium, 40 type 2 diabetic osteoporosis patients coexistent with PD, 50 type 2 diabetic osteoporosis patients with healthy periodontium, and 52 periodontally healthy individuals. miRNA expression measurements in the saliva were determined by real-time PCR.

**Results:**

The salivary expression of miR-25-3p was higher in type 2 diabetic osteoporosis patients than patients with T2DM only and healthy individuals (*P* < 0.05). Among type 2 diabetic osteoporosis patients, those with PD exhibited a higher salivary expression of miR-25-3p than those with healthy periodontium (*P* < 0.05). Among type 2 diabetic patients with healthy periodontium, a higher salivary expression of miR-25-3p was noted in those with OP than those without (*P* < 0.05). We also found a higher salivary expression of miR-25-3p in T2DM patients than healthy individuals (*P* < 0.05). It was revealed that the salivary expression of miR-25-3p was increased as the T scores of BMD of patients were lowered, the PPD and CAL values of patients were enhanced. The salivary expression of miR-25-3p used as a test to predict a diagnosis of PD among type 2 diabetic osteoporosis patients, a diagnosis of OP among type 2 diabetic patients, and a diagnosis of T2DM among healthy individuals produced AUC of 0.859. 0.824, and 0.886, respectively.

**Conclusion:**

The findings obtained from the study support salivary miR-25-3p confers non-invasive diagnostic potential for PD and OP among a cohort of elderly T2DM patients.

## Introduction

Diabetes mellitus (DM) represents a commonly serious metabolic disorder caused by either deficit in insulin secretion (type 1 diabetes mellitus, T1DM) or insulin resistance (type 2 diabetes mellitus, T2DM), among which type 2 diabetes mellitus (T2DM) accounts for approximately 90% of total cases of DM worldwide [1]. The incidence and prevalence of youth-onset T2DM is increasing rapidly with alarming trends, also including its complications [2]. There is growing evidence that T2DM may be associated with reduced bone mineral density (BMD) progression to osteoporosis (OP) and skeletal fragility [3]. Both T2DM and OP are affected by aging and quite often co-exist, especially in the elderly [4]. The older population suffering from T2DM co-existing with OP is more vulnerable to bone metabolic disorders in the oral cavity principally manifesting as periodontal disease (PD) [5]. PD has also been deemed as the sixth complication of T2DM, which is characterized by progressive destruction of tooth-supporting structure, loss of periodontal attachment, and resorption of alveolar bone [6, 7]. PD is a well-appreciated example of inflammation with pathogenic features and the current drug tacrolimus confers protection against the inflammation-induced tissue and bone loss in PD [8]. Interestingly, severe OP with reduced bone mineral content of the jaws may be associated with a less favorable attachment level in the cohort of postmenopausal women [9]. However, recent reviews of alveolar OP and worse periodontal status in the context of T2DM co-existing with OP together with the potential mechanisms of diabetic bone loss are limited, mainly centering on the adverse effects of OP alone on PD.

MicroRNAs (miRNAs) are known collectively as a group of non-coding RNAs that have been implicated in multiple human diseases, including T2DM [10], OP [11], and PD [12] by fine-tuning gene expression at the post-transcriptional level. The resulting studies showed significant changes in gingival crevicular fluid (GCF) miRNA expressions between PD and healthy subjects and these miRNA changes may be linked with inflammation response and differential expression of transglutaminases genes [13, 14]. As previous evidence showed, researchers compared the miRNA profiles of salivary exosome samples obtained from T2DM patients with those of healthy individuals and found that the salivary exosomal miR-25-3p expression was increased in T2DM patients, indicating their overexpression contributes to the development of T2DM-related PD [15]. miR-25-3p was also found to be highly enriched in bone marrow mesenchymal stem cells (BMSCs) derived from OP patients compared to normal BMSCs, suggesting miR-25-3p could inhibit osteogenic differentiation of BMSCs in the context of OP [16]. Of note, miR-25-3p was found to facilitate osteoclast activity via the modulation of nuclear factor I X expression, which may be a potential therapeutic target in osteoclasts of bone disorders in relation to impaired bone formation [17]. miR-25-3p was identified as a candidate regulator involving osteoblast and osteoclast differentiation processes for both type of osteoporosis by a large-scale screening based on microarray, qPCR validation and statistical algorithms [18]. However, the accuracy of miR-25-3p expression for the detection of OP and PD when compared to a mixed group of patients with T2DM remains to be elucidated. Herein, we propose an intriguing hypothesis that the salivary level of miR-25-3p could be considered as potential biomarkers for PD progression in type 2 diabetic osteoporosis patients. The purpose of the study is to determine the change of miR-25-3p expression in the saliva of type 2 diabetic osteoporosis patients coexistent with PD, with a focus on its ability to discriminate between type 2 diabetic osteoporosis patients coexistent with or without PD, between type 2 diabetic patients with or without osteoporosis, between diabetic and non-diabetic patients.

## Materials and methods

### Participants and eligibility

We prospectively recruited elderly patients with T2DM only, type 2 diabetic osteoporosis patients coexistent with or without PD, and healthy individuals who were admitted into the Second Affiliated Hospital of Xi’an Medical University for treatments or physical examinations between January 2021 and June 2022 to participate in the study. The diagnosis of T2DM was confirmed by the International Diabetes Federation (IDF) Diabetes Atlas 9th edition [19]. The diagnosis of osteoporosis was confirmed by T-score of BMD testing for the femoral neck, lumbar spine, total hip, or 1/3 radius [20]: T-scores not less than − 1.0 were deemed as normal BMD, T-scores ranging from − 1.0 to -2.5 as osteopenia and T-scores not more than − 2.5 as osteoporosis. Periodontal disease was diagnosed according to clinical and radiographic presentations with reference to the 2018 European Federation of Periodontology/American Academy of Periodontology (EFP/AAP) classification [21, 22], including interdental clinical attachment loss (CAL) of at least interproximal sites (not on the same tooth), or the buccal or oral CAL being more than 4 mm, with probing pocket depth (PPD) of equal or more than 4 mm at least four sites. The severity of PD was categorized based on interdental CAL at site of greatest loss, radiographic bone loss, and tooth loss, as previously reported [23]. Periodontally healthy conditions in this study were defined as absence of gingival inflammation (gingival index, GI = 0) along with PPD less than 3 mm in every site, CAL less than 2 mm, no clinical evidence of gingival inflammation, no radiographic evidence of alveolar bone loss (ABL), absence of loose teeth, and current use of dentures. The inclusion criteria were: (i) T2DM patients must have disease duration of at least two years and well-controlled diabetic conditions (HbA1c levels of < 8%); (ii) PD patients must be initially diagnosed with PD and have at least 16 natural teeth in their oral cavities; and (iii) aged ≥ 60 years. The exclusion criteria were: (i) current cigarette smokers; (ii) had any previous periodontal treatment, such as scaling and root planing treatment, in the last 6 months; (iii) use of immunosuppressants, antibiotics, or anti-inflammatory drugs in the last 6 months; (iv) any other systemic diseases: (v) history of SARS-CoV-2 infection; and (vi) dietary supplementations, pregnancy or breastfeeding.

### Salivary sample collection

The enrolled participants were requested to refrain from eating and drinking 1 h before saliva collection. At the beginning, they were asked to sit in a comfortable position and rinse their mouths with tab water for removal of any food debris, and then the unstimulated whole saliva (2–5 ml) was collected from each participant by using passive drool method, immediately placed into in a 50-mL sterile Falcon tube (Becton, Dickinson and Company, New Jersey, USA). The collected saliva was centrifuged (2500 g for 10 min) at room temperature. Supernatant was isolated and stored at -80ºC until further analyzed.

### miRNA expression measurements

Extraction of total RNA applied the TRIzol reagent (Invitrogen, USA). The synthesis of cDNA applied the TaqMan miRNA Reverse Transcription kit (Invitrogen, USA). The quantification of hsa-miR-25-3p was performed using TaqMan MicroRNA Assays (Applied Biosystems, USA) with the aid of the LightCyclerⓇ 480 Real-Time PCR System (Roche) according to the thermocycler protocols: 95 °C for 5 min plus 40 amplification cycles of 94 °C for 30 s, 62 °C for 30 s, and 72 °C for 30 s. All the primer sequences are available in Table 1. Each sample was tested in duplicate. The cycle threshold (Ct) values were normalized to the level of U6 and results were then converted into fold change using the 2^−ΔΔCt^ formula.

**Table 1:** The primer sequences used in the real-time PCR.

Primer		Sequence (5’-3’)
hsa-miR-25-3p	RT primer	5’-CTCAACTGGTGTCGTGGAGTCGGCAATTCAGTTGAGtcagaccg-3’
	PCR primer (forward)	5’-GCCGAGCAUUGCACUUGUCU-3’
U6	PCR primer (forward)	5’-CCTCGCTTCGGCAGCACATATAC-3’
Universal reverse primer		Provided by Applied Biosystems

### Clinical outcome variables

Each participant was evaluated for their BMD and periodontal indicators including PPD, CAL, bleeding index (BI), and periodontal plaque index (PLI) after admission; (i) BMD: BMD measurements of the femoral neck, lumbar spine, total hip, and 1/3 radius applied the DPX-MD dual energy X-ray bone densitometry (LUNA, USA); (ii) PPD: the six sites (buccal/lingual mesial, central, distal) of each tooth were examined with periodontal probe, that is, the distance from the gingival margin to the bottom of the gingival sulcus or periodontal pocket; (iii) CAL: when gingival retraction occurs, CAL equals to PPD plus the distance from enamel cementum boundary to gingival margin; when there is no gingival retraction, CAL equals to PPD out of the distance from enamel bone to gingival margin; (iv) BI: the gingival sulcus or periodontal pocket of the participants was gently probed with a blunt probe. After removing the probe, 30 s later, the gingival bleeding of the participants was observed; absence of bleeding and inflammation in the gingiva was scored 0 point; slight evidence of inflammatory lesions in the gingiva without bleeding was scored 1; evidence of inflammatory lesions in the gingiva with spot bleeding was scored 2 points; evidence of inflammatory lesions in the gingiva with bleeding after probing and blood spreading along the gingival margin was scored 3 points; evidence of inflammatory lesions in the gingiva with bleeding after probing and blood overflowing the gingival sulcus was scored 4 points; presence of spontaneous bleeding of gingiva was scored 5 points. (v) PLI: absence of plaque in the gingival margin area was scored 0 point; no plaque by visual inspection but evidence of plaque by the probe tip was scored 1 point; moderate plaque in the gingival margin area was scored 2 points; a large number of plaques in gingival margin area and maxillofacial area was scored 3 points. The periodontal indicators of each participant were evaluated and recorded by the well-experienced periodontist. The clinical measurements were recorded and the saliva samples were collected from the sampling site at baseline.

### Statistical analysis

The outcomes were described using mean ± standard deviation for measurement variables, using counts and percentages for categorical variables. The chi-square test, unpaired t test, and one-way analysis of variance (ANOVA) (with post-hoc tests) were used for statistical comparison after the data were verified for the normal distribution and the homogeneity of variance. Pearson correlation test were used to assess the association between the salivary expression of miR-25-3p, and periodontal indicators. Area under the curve (AUC) using receiver operating characteristic (ROC) method with cut-off points identified for the highest sensitivity and specificity as assessed by Youden’s index were used to estimate the diagnostic value of miR-25-3p for type 2 diabetic osteoporosis coexistent with or without PD. Calculations and figure visualization was performed GraphPad Prism 8 (GraphPad Software, USA), with the possibility less than 0.05 (*P* < 0.05) used to reflect the presence of significant difference.

## Results

### Demographics and clinical characteristics of subjects

According to the inclusion and exclusion criteria, we finally recruited 45 patients with T2DM only (T2DM group), 40 type 2 diabetic osteoporosis patients coexistent with PD (T2DM/OP + PD group), 50 type 2 diabetic osteoporosis patients with periodontally healthy conditions (T2DM/OP group), and 52 healthy individuals (normal group). Their demographics and clinical characteristics are listed in Table 2. No significant difference was noted regarding sex, age, body mass index (BMI), antidiabetic treatment, and the proportion of SARS-CoV-2 vaccinators among T2DM, T2DM/OP + PD, T2DM/OP, and normal groups (*P* > 0.05). Two groups of patients with osteoporosis exhibited significantly different T scores of BMD compared to two groups of patients without osteoporosis. There was no significant difference on the T scores of BMD between T2DM/OP + PD and T2DM/OP, between T2DM and normal groups (*P* > 0.05). The PPD, CAL, BI, and PLI at the baseline were statistically greater in the T2DM/OP + PD groups than periodontally healthy groups (T2DM, T2DM/OP, and normal groups) (*P* < 0.05).

**Table 2:** Demographics and clinical characteristics of the participants at the baseline

Variable	T2DM (n = 45)	T2DM/OP + PD (n = 40)	T2DM/OP (n = 50)	Normal (n = 52)	*P*
Sex/male (%)	28 (62.22%)	24 (60.00%)	29 (58.00%)	31 (59.62%)	0.981
Age (year)	69.13 ± 3.62	69.23 ± 5.78	69.40 ± 4.25	69.00 ± 5.25	0.979
BMI	26.68 ± 2.67	26.23 ± 2.04	26.02 ± 2.69	27.00 ± 2.09	0.174
Glycemic control					0.448
Metformin with alogliptin	18 (40.00%)	14 (35.00%)	24 (48.00%)	/	
Metformin with glimepiride	27 (60.00%)	26 (65.00%)	26 (52.00%)	/	
SARS-CoV-2 vaccinators	32 (71.11%)	29 (72.50%)	37 (74.00%)	40 (76.92%)	0.926
T-score of BMD	0.20 ± 0.72^*#^	-2.81 ± 0.22	-2.77 ± 0.20	0.31 ± 0.51^*#^	
PPD (mm)	1.90 ± 0.29^*^	4.55 ± 0.22	1.89 ± 0.34^*^	1.81 ± 0.26^*^	
CAL (mm)	0.55 ± 0.30^*^	5.58 ± 0.21	0.57 ± 0.29^*^	0.46 ± 0.35^*^	
BI	1.81 ± 0.24^*^	3.64 ± 0.45	1.79 ± 0.29^*^	1.73 ± 0.20^*^	
PLI	0.40 ± 0.23^*^	2.26 ± 0.33	0.38 ± 0.20^*^	0.32 ± 0.20^*^	

BMI, body mass index; PPD, probing pocket depth; CAL, clinical attachment level; BI, bleeding index; *P* was yielded by the chi-square test and one-way ANOVA; *P < 0.05 (by unpaired t test) compared to T2DM/OP + PD; # *P* < 0.05 (by unpaired t test) compared to T2DM/OP group

### The salivary expression of mir-25-3p in relation to PD occurrence in type 2 diabetic osteoporosis

**Fig. 1:**
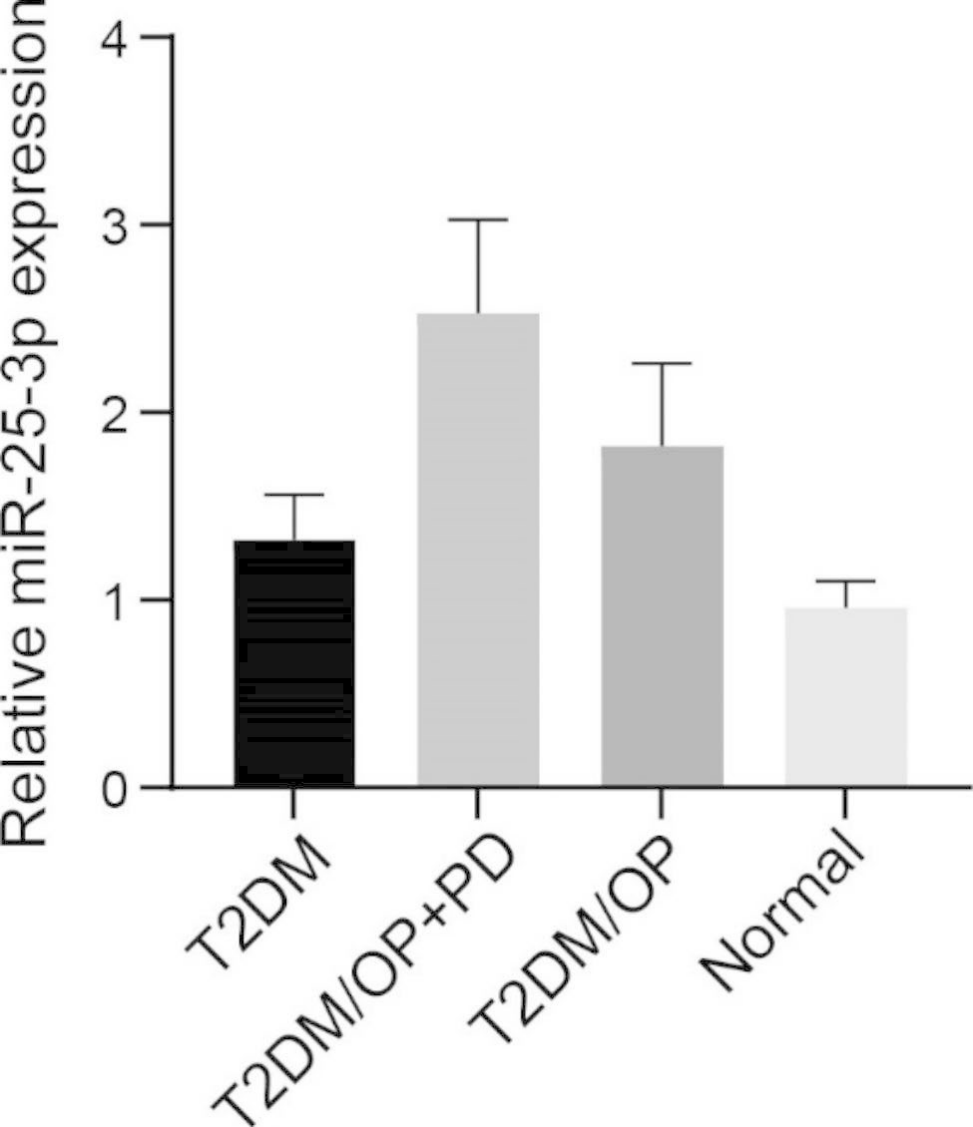
The salivary expressions of miR-25-3p among T2DM, T2DM/OP + PD, T2DM/OP, and normal groups were determined by real-time PCR. The one-way ANOVA followed by Tukey’s post hoc test was used for statistical analysis

The salivary expression of miR-25-3p exhibited significant differences among T2DM, T2DM/OP + PD, T2DM/OP, and normal groups (F = 165.6 and *P* < 0.001, Fig. 1). More specifically, it was found that the salivary expressions of miR-25-3p were higher in the T2DM/OP + PD and T2DM/OP groups both than T2DM and normal groups (*P* < 0.001). More specifically, the patients in the T2DM/OP + PD group exhibited a higher salivary expression of miR-25-3p than those in the T2DM/OP group (*P* < 0.001), which indicated the change of salivary miR-25-3p expression due to PD occurrence among type 2 diabetic osteoporosis patients. In addition, a higher salivary expression of miR-25-3p was noted in the T2DM/OP group than the T2DM group, reflecting the salivary expression of miR-25-3p may be affected by OP among type 2 diabetic individuals (*P* < 0.001). We also found a higher salivary expression of miR-25-3p in the T2DM group than the normal group (*P* < 0.001), which revealed the alternation of salivary miR-25-3p expression due to the development of T2DM.

### Associations between salivary expressions of miR-25-3p, BMD, and periodontal indicators

**Fig. 2:**
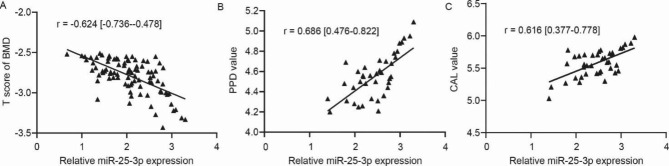
Associations between salivary expression of miR-25-3p, BMD, and periodontal indicators. (**A**), miR-25-3p and T score of BMD. (**B**), miR-25-3p and PPD value. (**C**), miR-25-3p and CAL value

Considering the change of salivary miR-25-3p expression due to PD and OP among type 2 diabetic patients, we were interested in evaluating the associations between salivary expressions of miR-25-3p, BMD, and periodontal indicators. Results showed that, among type 2 diabetic osteoporosis patients either with or without PD, the salivary miR-25-3p expression was increased as the T scores of BMD of patients were lowered (*P* < 0.001, Fig. 2A). Among type 2 diabetic osteoporosis patients with PD, the salivary miR-25-3p expression was increased as the PPD and CAL values of patients were enhanced (*P* < 0.001, Fig. 2B, C), while it failed to show correlations with other periodontal indicators (*P* > 0.05).

### The diagnostic performance of mir-25-3p in type 2 diabetic osteoporosis patients co-existing with PD

**Fig. 3:**
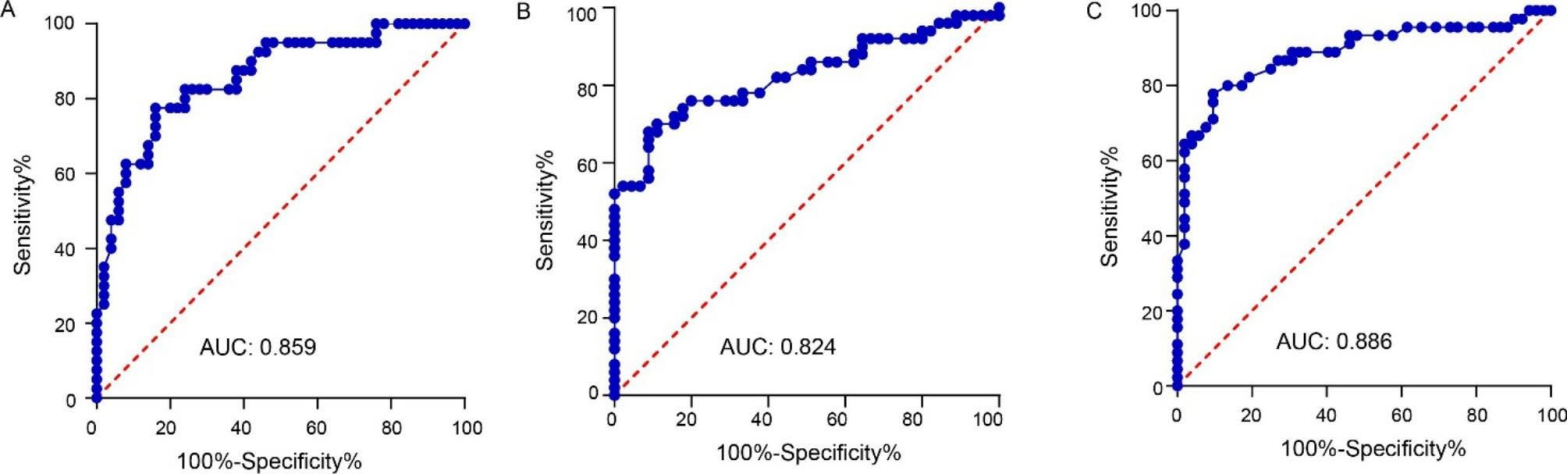
The ROC method was used to evaluate the salivary expression of miR-25-3p in its ability to discriminate between type 2 diabetic osteoporosis patients coexistent with or without PD (**A**), between type 2 diabetic patients with or without osteoporosis (**B**), between diabetic and non-diabetic patients (**C**)

We applied the ROC method to rank the salivary expression of miR-25-3p in its ability to discriminate between type 2 diabetic osteoporosis patients coexistent with or without PD, between type 2 diabetic patients with or without osteoporosis, between diabetic and non-diabetic patients. Larger AUC reflects better diagnostic performance exerted by miR-25-3p on PD, OP, and/or T2DM. As shown by Fig. 3A, miR-25-3p used as a test to predict a diagnosis of PD among type 2 diabetic osteoporosis patients produced an AUC of 0.859 (sensitivity: 77.50%; specificity: 84.00%). As shown by Fig. 3B, miR-25-3p used as a test to predict a diagnosis of OP among type 2 diabetic patients yielded an AUC of 0.824 (sensitivity: 68.00%; specificity: 91.11%). As shown by Fig. 3C, miR-25-3p used as a test to predict a diagnosis of T2DM among healthy individuals generated an AUC of 0.886 (sensitivity: 77.78%; specificity: 90.38%).

### Putative target genes of mir-25-3p in type 2 diabetic osteoporosis patients co-existing with PD

**Fig. 4:**
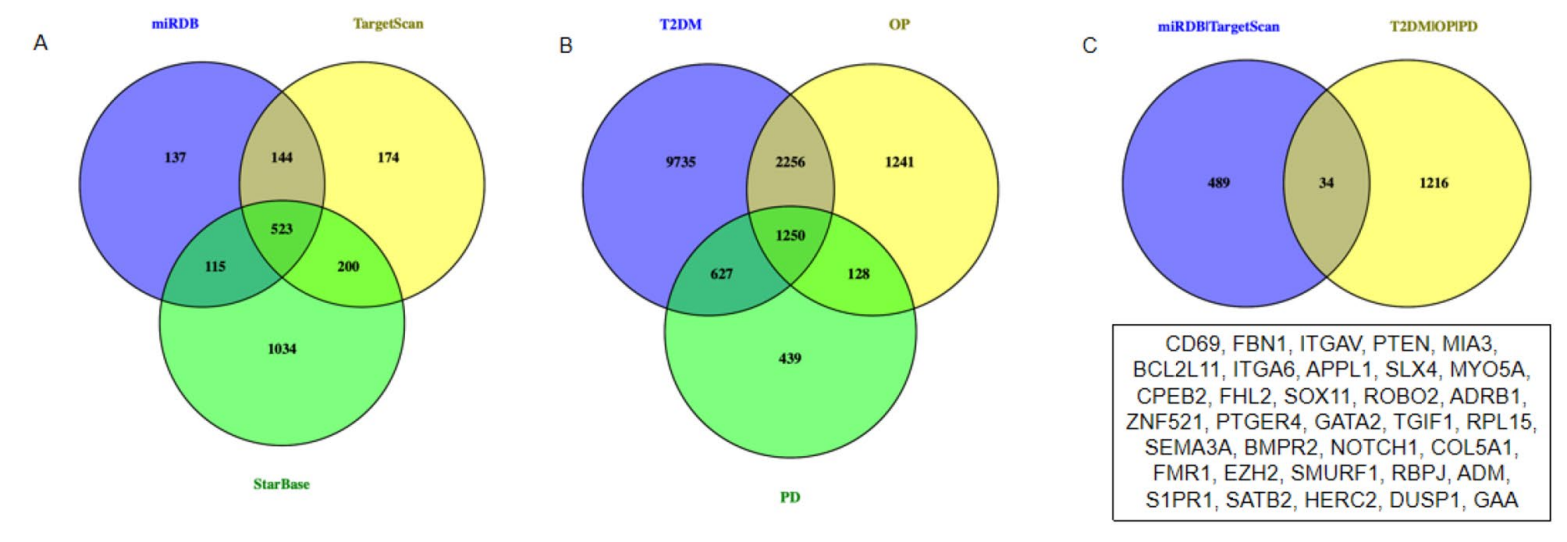
Putative target genes of miR-25-3p, disease target genes related to PD, OP, and T2DM. (**A**), 523 common genes targeted by miR-25-3p among the Starbase, TargetScan, and miRDB databases. (**B**), 1250 genes related to three of PD, OP, and T2DM by analyzing the GeneCards database. (**C**), 34 genes targeted by miR-25-3p and related to PD, OP, and T2DM together

We used Starbase (https://starbase.sysu.edu.cn/starbase2/index.php), TargetScan (https://www.targetscan.org/vert_80/), miRDB (http://mirdb.org/mirdb/index.html), and obtained 523 genes targeted by miR-25-3p (Fig. 4A). Next, we searched the GeneCards database (https://www.genecards.org/) and obtained 1250 genes related to PD, OP, and T2DM (Fig. 4B). Between 523 genes and 1250 genes, we found 34 overlapping genes (Fig. 4C).

## Discussion

Older adults among T2DM sufferers have an increased risk of developing diseases related to bone loss [24]. There is increasing evidence of miRNAs served as diagnostic and surrogative biomarkers for T2DM and its complications owing to their high stability, sensitivity, and less-invasive property [25]. This study was performed with the purpose to investigate the expression patterns of miR-25-3p in the saliva of elderly T2DM patients when OP and PD co-existed, thereby evaluating its clinical diagnostic value. The present study provided evidence that the salivary level of miR-25-3p could be considered as potential biomarkers for PD progression in type 2 diabetic osteoporosis patients.

Clinical characteristics showed obvious higher levels of periodontal indicators at baseline, including PPD, CAL, BI, and PLI, in type 2 diabetic osteoporosis patients co-existing with PD than periodontally healthy subjects. Although no remarkable differences were noted regarding periodontal indicators among included subjects with healthy periodontium, their levels were slightly higher in type 2 diabetic patients than healthy individuals. T2DM may aggravate the severity of PD, which was consistent with previous studies, indicating T2DM patients were more likely to suffer from severe periodontitis [26]. Emerging technologies to amplify and detect nucleic acids in the body fluid have allowed for sensitive methods to link gene expression profiling with specific states of disease [27]. Usually, tissue biopsy and histopathological analyses are the gold standard methods for the diagnosis of oral cancers. However, it is often not possible to perform tissue biopsy and histopathological analyses for bone or oral cavity diseases. Salivary samples or liquid biopsy samples serving as novel minimally invasive tools is fundamental for molecular investigations for studies of oral diseases, such as circulating tumor DNA (ctDNA), miRNAs, proteins, and exosomes [28, 29]. Saliva as oral fluid is essential to oral biofilm formation and host defense, and has been used to evaluate the progression of PD [30] and as non-invasive diagnostic tool for insulin-resistance [31]. In addition to salivary samples, GCF samples are regarded as important liquid biopsy samples for PD analysis, and GCF miRNAs profiles of PD patients showed higher levels of miRNA 7a-5p, miRNA 21-3p, miRNA 21-5p, miRNA 200b-3p, and miRNA 200b-5p levels, lower miRNA 100-5p and miRNA 125-5p levels [13]. Byun et al. demonstrated that salivary miR-25-3p expression was significantly enriched in obese patients with T2DM compared with T2DM or healthy individuals, and their animal study confirmed positive effects of down-regulated miR-25-3p on mice with periodontitis [15]. miR-25-3p has been reported to directly reduce insulin expression through transcriptional regulation of β-cell specific genes [32]. The present study further compared miR-25-3p expression in the saliva of type 2 diabetic osteoporosis patients with T2DM patients without OP and found a significant miR-25-3p increase in type 2 diabetic osteoporosis patients. The inhibitory role of miR-25-3p on osteogenic differentiation of BMSCs during OP was previously demonstrated, reflecting an increasing expression of miR-25-3p may be associated with bone loss [16]. These findings were also supported by larger AUC reflecting better diagnostic performance exerted by miR-25-3p on PD, OP, and/or T2DM, suggesting that the changes of miR-25-3p expression in the saliva were induced by T2DM and more evident due to the occurrence of OP and PD.

Identifying target genes by post-transcriptional regulation of miRNAs is a key strategy to understand biological processes of miRNAs involved in the development of diseases [33]. Our study identified that a total of 34 target genes may be implicated in the development of PD, OP, and T2DM together, including CD69, FBN1, ITGAV, PTEN, MIA3, BCL2L11, ITGA6, APPL1, SLX4, MYO5A, CPEB2, FHL2, SOX11, ROBO2, ADRB1, ZNF521, PTGER4, GATA2, TGIF1, RPL15, SEMA3A, BMPR2, NOTCH1, COL5A1, FMR1, EZH2, SMURF1, RBPJ, ADM, S1PR1, SATB2, HERC2, DUSP1, and GAA. However, the alterations of most of these genes and their actual interactions involved in the pathophysiology of T2DM progression to coexistence with OP and PD remain to be elucidated.

Several limitations of this study should be noted when the data are interpretated: (i) an unbiased screening is more appropriate for a first screening, which requires high-throughput sequencing of salivary samples collected from T2DM complicated with OP and/or PD; (ii) lack of a validation cohort for testing diagnostic performance; (iii) general limitations with biomarkers studies in analysis of circulating miRNAs, such as relatively high protein and lipid content of these body fluids can interfere with RNA isolation and the current lack of unequivocally accepted normalization strategies for human circulating miRNAs qRT-PCR-based analysis; and (iv) although qPCR still represents the conventional method for the analysis of liquid biopsy samples including saliva samples, further validations by using digital PCR, droplet digital PCR, mass spectrometry, and next generation sequencing to obtain greater accuracy of such analyses will be required.

In conclusion, the miR-25-3p expression may be induced by T2DM and increased as the disease progression to coexistence with OP and PD. The present study supports that salivary miR-25-3p confers non-invasive diagnostic potential for PD and OP among a cohort of elderly T2DM patients. In the future, salivary samples collected from T2DM complicated with OP and/or PD should be submitted to perform gene expression profiling by high-throughput sequencing, in a bid to confirm the feasibility of functional studies centering to miR-25-3p control of target genes.

## Data Availability

All data generated or analyzed during this study are included in this published article.
